# Supplementary material for: Sex differences in symptoms following the administration of BNT162b2 mRNA COVID-19 vaccine in children below 5 years of age in Germany (CoVacU5): a retrospective cohort study
Source: Biol Sex Differ. 2024 Sep 26;15:74. doi: 10.1186/s13293-024-00651-x (PMC11426002; doi:10.1186/s13293-024-00651-x)
Supplement: Supplementary file 1 — Supplementary Material 1 [file 13293_2024_651_MOESM1_ESM.docx]

Supplemental Table 1. Post-vaccination symptoms after BNT162b2 vaccine, n/N (%)

|  |  | First Vaccination | | | Second Vaccination | | | Third Vaccination | | |
| --- | --- | --- | --- | --- | --- | --- | --- | --- | --- | --- |
| Dose | Symptoms | Female | Male | p-val^a^ | Female | Male | p-val^a^ | Female | Male | p-val^a^ |
| 3µg | Local | 286/883 (32.4) | 252/874 (28.8) | 0.4233 | 145/588 (24.7) | 153/574 (26.7) | >0.999 | 3/26 (11.5) | 9/26 (34.6) | 0.1931 |
|  | General | 92/881 (10.4) | 94/868 (10.8) | >0.999 | 69/587 (11.8) | 55/569 (9.7) | >0.999 | 1/26 (3.8) | 2/26 (7.7) | >0.999^b^ |
|  | Fever | 23/881 (2.6) | 27/868 (3.1) | >0.999 | 24/587 (4.1) | 22/569 (3.9) | >0.999 | 0/26 (0) | 0/26 (0) | - |
|  | Musculosk | 11/877 (1.3) | 13/868 (1.5) | >0.999 | 8/584 (1.4) | 8/571 (1.4) | >0.999 | 0/25 (0) | 0/26 (0) | - |
| 5µg | Local | 522/1511 (34.5) | 461/1590 (29) | **0.0036** | 423/1324 (31.9) | 388/1401 (27.7) | 0.0608 | 38/107 (35.5) | 39/108 (36.1) | >0.999 |
|  | General | 203/1508 (13.5) | 194/1589 (12.2) | >0.999 | 142/1322 (10.7) | 151/1400 (10.8) | >0.999 | 10/107 (9.3) | 14/108 (13) | >0.999 |
|  | Fever | 65/1508 (4.3) | 60/1589 (3.8) | >0.999 | 56/1322 (4.2) | 60/1400 (4.3) | >0.999 | 8/107 (7.5) | 4/108 (3.7) | 0.9129^b^ |
|  | Musculosk | 39/1505 (2.6) | 39/1586 (2.5) | >0.999 | 28/1319 (2.1) | 35/1396 (2.5) | >0.999 | 5/107 (4.7) | 1/108 (0.9) | 0.3814^b^ |
| 10µg | Local | 548/1111 (49.3) | 504/1217 (41.4) | **0.0005** | 569/1308 (43.5) | 493/1374 (35.9) | **0.0002** | 92/258 (35.7) | 112/267 (41.9) | 0.5578 |
|  | General | 142/1106 (12.8) | 143/1214 (11.8) | >0.999 | 150/1304 (11.5) | 132/1372 (9.6) | 0.4519 | 37/258 (14.3) | 31/267 (11.6) | >0.999 |
|  | Fever | 23/1106 (2.1) | 44/1214 (3.6) | 0.1059 | 54/1304 (4.1) | 43/1372 (3.1) | 0.6544 | 16/258 (6.2) | 13/267 (4.9) | >0.999 |
|  | Musculosk | 44/1105 (4) | 47/1208 (3.9) | >0.999 | 59/1302 (4.5) | 38/1366 (2.8) | 0.0632 | 7/258 (2.7) | 15/264 (5.7) | 0.3658 |

^a^ Chi-square test unless otherwise stated and adjusted for multiple testing by Bonferroni correction. ^b^Fisher’s exact test
