# Supplementary material for: Sex differences in symptoms following the administration of BNT162b2 mRNA COVID-19 vaccine in children below 5 years of age in Germany (CoVacU5): a retrospective cohort study
Source: Biol Sex Differ. 2024 Sep 26;15:74. doi: 10.1186/s13293-024-00651-x (PMC11426002; doi:10.1186/s13293-024-00651-x)
Supplement: Supplementary file 2 — Supplementary Material 2 [file 13293_2024_651_MOESM2_ESM.docx]

Supplemental Table 2. Non-BNT162b2 vaccines administered since January 15^th^ 2022, n (%)

|  | All | Female | Male |  |
| --- | --- | --- | --- | --- |
|  | n=4570 (100) | n=2251 (49.3) | n=2319 (50.7) | p-val^a^ |
| Non-BNT162b2 vaccines (yes) | 1490 (32.6) | 718 (31.9) | 772 (33.3) | >0.999 |
| Influenza | 540 (11.8) | 254 (11.3) | 286 (12.3) | >0.999 |
| Meningococcal | 476 (10.4) | 245 (10.9) | 231 (10) | >0.999 |
| Measles/mumps/rubella with/without chickenpox | 387 (8.5) | 195 (8.7) | 192 (8.3) | >0.999 |
| Tetanus/diphtheria/pertussis and/or pediatric polio | 364 (8) | 181 (8) | 183 (7.9) | 0.279 |
| Hepatitis A/B | 159 (3.5) | 71 (3.2) | 88 (3.8) | >0.999 |
| Human papillomavirus | 4 (0.1) | 1 (0) | 3 (0.1) | >0.999 |
| Other | 266 (5.8) | 127 (5.6) | 139 (6) | >0.999 |

^a^Adjusted for multiple testing by Bonferroni correction.
